# Capacitive Sensors and Actuators by CMOS MEMS Foundry

**DOI:** 10.3390/mi17060732

**Published:** 2026-06-17

**Authors:** Lung-Jieh Yang, Chandrashekhar Tasupalli, Wei-Chen Wang, Yi-Jen Wang, Valliammai Muthuraman, Chi-Yuan Lee

**Affiliations:** 1 Department of Mechanical and Electromechanical Engineering, Tamkang University, New Taipei City 251391, Taiwan; chandrasekharamma25@gmail.com (C.T.); bmw001234567@gmail.com (W.-C.W.); 2 Taiwan Semiconductor Research Institute, National Institute of Applied Research Laboratories (NARLabs), Hsinchu 300091, Taiwan; nick.wang@niar.org.tw; 3 Department of Electronics and Communication Engineering, Vel Tech Rangarajan Dr. Sagunthala R&D Institute of Science and Technology, Chennai 600062, Tamil Nadu, India; drvalliammaim@veltech.edu.in; 4 Department of Mechanical Engineering, Yuan Ze Fuel Cell Center, Yuan Ze University, Taoyuan 320, Taiwan; cylee@saturn.yzu.edu.tw

**Keywords:** complementary-metal-oxide-semiconductor (CMOS) MEMS, fabless foundry, capacitive sensors, electrostatic actuators, post-process, micro mirror

## Abstract

This article introduces the current status of the 0.18-micron CMOS MEMS foundry service platform provided by the Taiwan Semiconductor Research Institute (TSRI), extensively covering the CMOS MEMS components that it has supported in development and fabrication. It also attempts to expand the foundry service scope to the broader categories of capacitive sensors and electrostatic actuators. On the one hand, for fabless MEMS component designers, TSRI currently directly allows the design of two types of components: flow sensors with uniformly perforated membranes and actuators with comb-shaped interdigital electrodes. This service also includes tape-out and wire bonding packaging procedures, following procedures similar to those used by general IC designers. On the other hand, this article specifically presents a clear and feasible approach for MEMS designers equipped with simple wet-etching facilities and a clear and feasible approach to develop further CMOS MEMS components such as capacitive pressure sensors, accelerometers, micro mirrors, and scratch drive actuators with minimal post-processing and chip packaging steps. This work provides a practical CMOS-MEMS design and post-processing guideline for extending the current TSRI foundry platform toward capacitive sensing and electrostatic actuation applications with minimal additional fabrication complexity.

## 1. Introduction

The development of Taiwan’s semiconductor industry and its supply chain, represented by TSMC, has attracted international attention in recent years due to its market share of over 90% in advanced chips, and it has even become a strategic battleground in international geopolitics [1,2]. Whether advanced-node or mature-node semiconductor chips, the current commercial transistor manufacturing process is based on complementary metal-oxide-semiconductor (CMOS) technology. Even when delving into the most advanced gate-all-around (GAA) 2 nm process node [3], it still cannot escape the scope of CMOS technology.

Compared to the high-end nanometer process node CMOS circuits, the CMOS MEMS technology capable of producing sensors and actuators is rarely scaled down below the 180-nanometer process node. For example, based on the chip foundry services provided by the Taiwan Semiconductor Research Institute (TSRI) in 2025 [4], United Microelectronics Corp. (UMC) and TSMC offer a 0.18 µm CMOS MEMS process, which seems to be sufficient for MEMS researchers’ education and training as well as for the development of innovative MEMS components. In fact, after the UMC and TSMC’s standard CMOS processes, it takes about a month for TSRI to continue completing the MEMS post-process, wafer dicing, bonding, and packaging. These value-added services beyond foundry tape-out include reactive ion etching (RIE) to open holes in the approximately 11 μm thick silicon oxide layer of CMOS, as well as subsequent silicon substrate cavity etching beneath the so-called MEMS_OPEN areas. The latter etching is believed to be done by using XeF_2_ silicon gaseous-phase etching [5].

**Figure 1:**
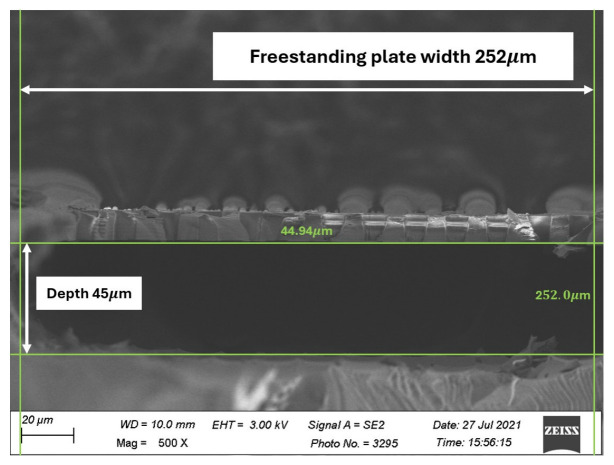
The 45 μm-deep cavity underneath the MEMS_OPEN area after the CMOS MEMS post-process provided by TSRI [6].

The implementation results in the literature [6,7] show that the CMOS MEMS post-process provided by TSRI can achieve a silicon substrate etching depth of approximately 45 μm under the MEMS_OPEN area, effectively suspending CMOS MEMS microstructures, as shown in Figure 1. Therefore, chip designers do not even need to add any post-processing themselves, nor do they need to have their own expensive cleanroom facilities and process equipment. This allows rapid entry into the testing phase after receiving the fabricated CMOS chips, shortening the component development cycle time. On a related note, the CMOS MEMS post-process provided by TSRI, including the previous UMC and TSMC 0.18 μm processes, both use 8-inch wafer process equipment. As for CMOS foundry processes with linewidths smaller than 0.18 μm, they are both made in UMC and TSMC 12-inch wafer factories. But there may be no corresponding RIE/XeF_2_ process equipment for MEMS post-processing the 12-inch wafers. Therefore, the CMOS MEMS post-process is not yet provided for CMOS processes with linewidths below 0.18 μm.

The following introduces the CMOS MEMS foundry provided by TSRI. TSRI (formerly known as Chip Implementation Center—CIC) has been collaborating with UMC and TSMC since the 1990s to provide 0.35 μm CMOS standard foundry services, offering IC design training and innovative component development for the Taiwanese academic community. At first, MEMS researchers at National Taiwan University (NTU) and National Chung Hsing University (NCHU) widely developed different kinds of MEMS devices including accelerometers [8], test keys [9,10], microwave switches [11], band pass filters [12], pressure sensors [13], humidity sensors [14], gas sensors [15], and micro generators [16].

After 2000, close collaboration among TSRI, industries, and CMOS foundries has enabled the development of advanced CMOS-MEMS platforms not only at NTU/NCHU in Taiwan [17,18] but also at National Tsing Hua University (NTHU) and National Yang Ming Chiao Tung University (NYCU) in Hsinchu. More studies have further reinforced the concept of CMOS-MEMS as a versatile platform for multi-functional sensing systems. Fang et al. in NTHU provided a comprehensive overview of CMOS-MEMS technology, demonstrating that standard CMOS processes combined with post-CMOS micromachining enable the fabrication of a wide range of sensors, including accelerometers, pressure sensors, tactile sensors, and infrared sensors [19]. This highlights the capability of CMOS-MEMS to integrate multiple sensing functions within a single chip, forming compact and highly integrated sensing systems. However, at that time the MEMS researchers/designers had to handle the MEMS post-processing fully on their own.

Since 2012, CIC has begun providing a 0.18 μm CMOS MEMS process design guide and updated foundry services. Significant progress in CMOS-MEMS technology started to be achieved within the Taiwan semiconductor ecosystem. Tseng et al. also introduced a comprehensive CMOS-MEMS design and fabrication platform that integrates standard CMOS processes with post-CMOS micromachining techniques, enabling the realization of various MEMS devices through a unified and reliable workflow [20]. These platforms demonstrate that suspended microstructures can be fabricated using multilayer CMOS structures followed by controlled dry etching and silicon release processes owned by the MEMS designers, which are consistent with modern CMOS-MEMS foundry services [21,22].

In terms of device development, Hsieh et al. reported a CMOS-MEMS capacitive tactile sensor with a vertically integrated sensing electrode array, which significantly enhances sensitivity by utilizing stacked CMOS metal layers as sensing structures [23]. This device highlights the advantage of CMOS-MEMS technology in achieving compact size, high integration, and improved sensing performance, making it suitable for applications such as robotics and human–machine interfaces. Similarly, Lee et al. presented CMOS-MEMS technologies for environmental sensing systems, including infrared, pressure, and gas sensors, demonstrating the capability of CMOS-MEMS platforms to support multi-functional sensing hubs with low power consumption and high system integration [24].

These studies collectively demonstrate that CMOS-MEMS technology in Taiwan has evolved into a more mature and versatile platform capable of realizing diverse sensing devices. By combining standard CMOS fabrication with post-CMOS release processes, such as dry etching and substrate removal, CMOS-MEMS devices can achieve high performance, strong integration, and scalable manufacturing. This development is closely aligned with the TSRI CMOS-MEMS post-processing approach, where standardized release techniques enable reliable fabrication of suspended microstructures for capacitive sensing and actuation applications.

**Figure 2:**
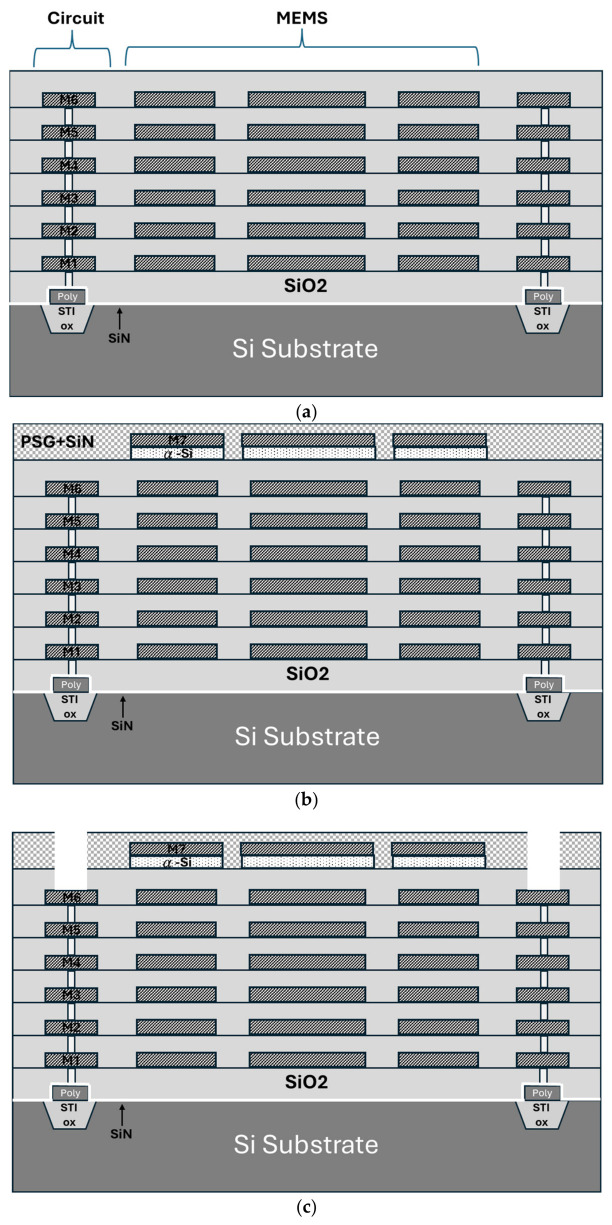

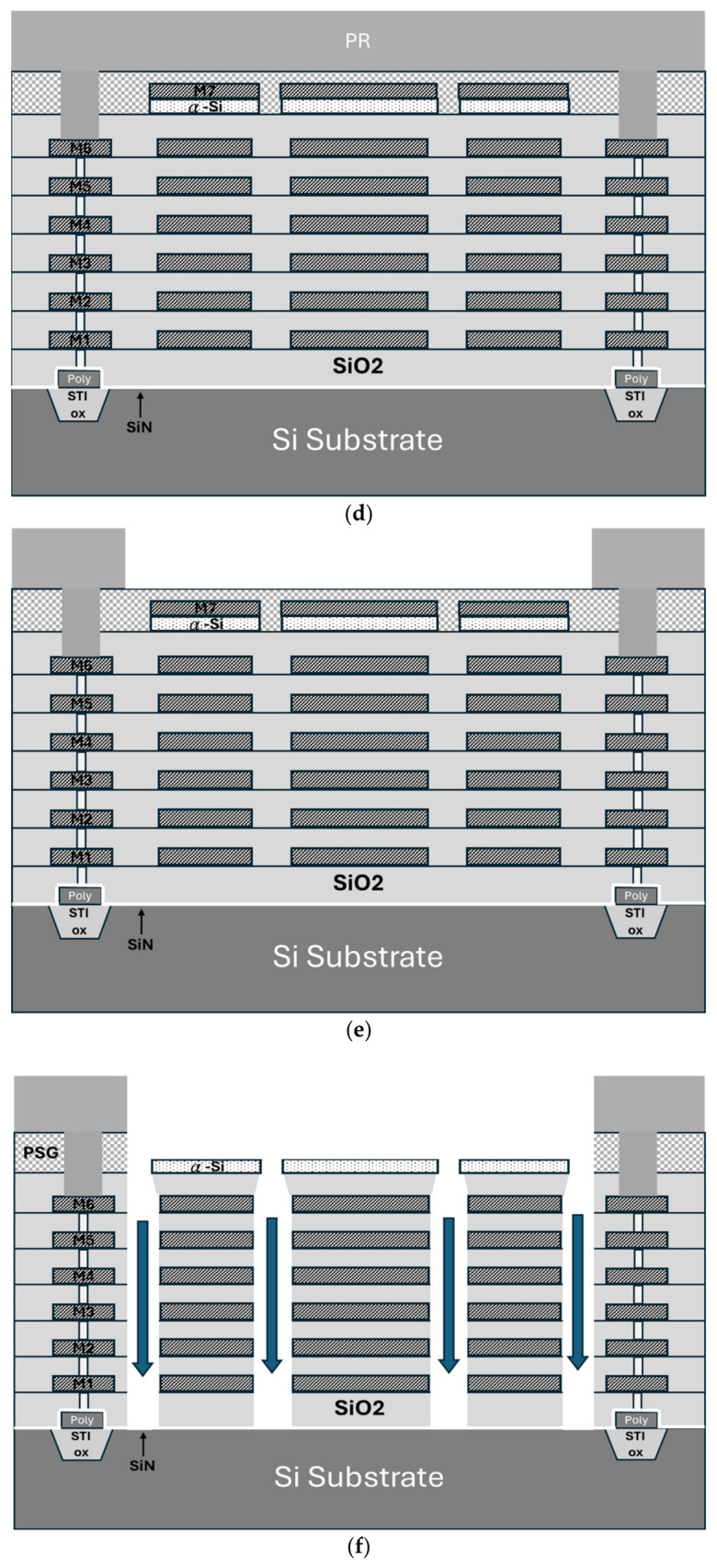

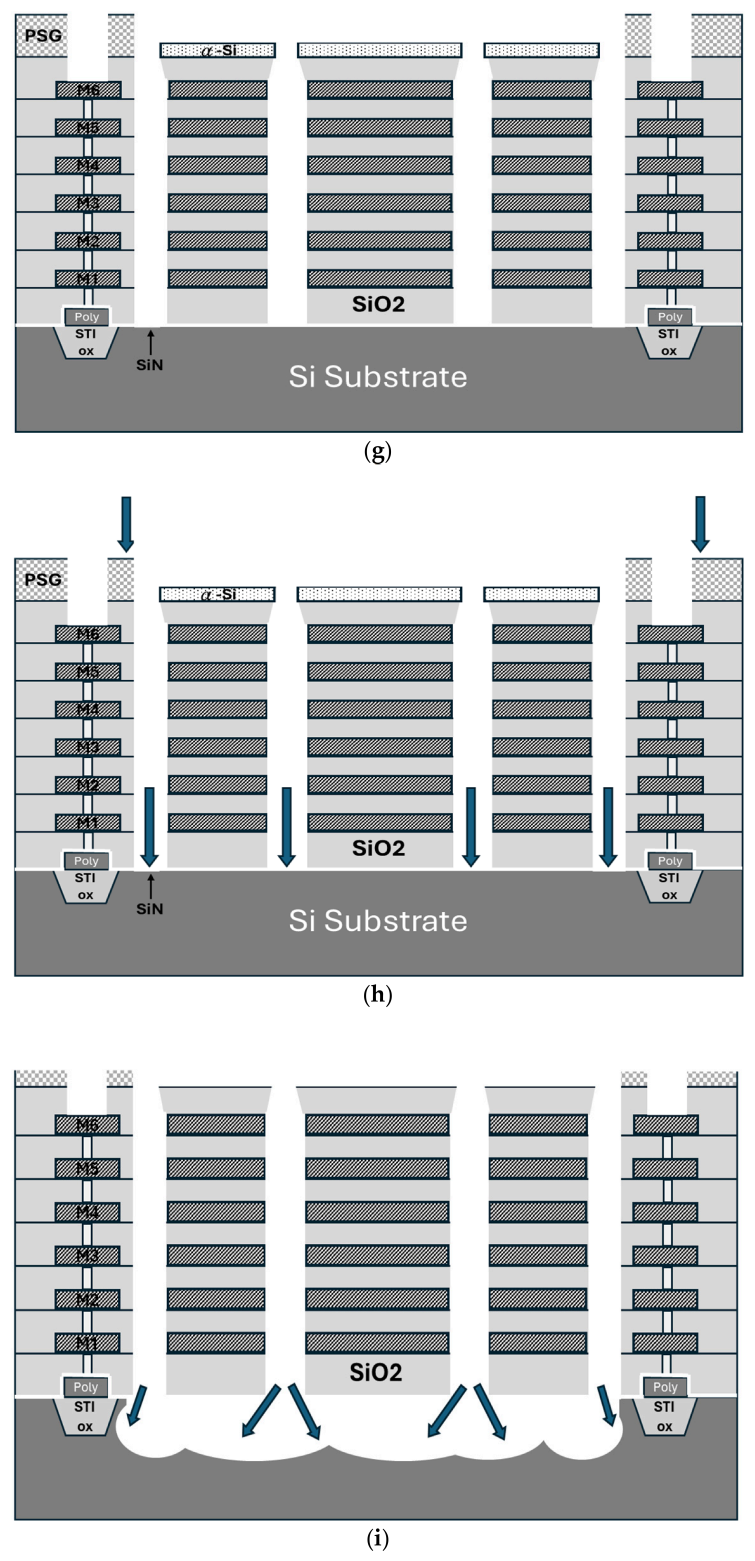
The process flow of TSRI CMOS MEMS foundry [4]: (**a**) the cross-sectional view of the standard 1P6M CMOS layers; MEMS means the MEMS_OPEN region with release microstructures finally. (**b**) The cross-sectional view of the CMOS layers with an additional patterned amorphous silicon (α-Si), metal layer (ME7 mask is needed) and passivation layer. (**c**) The cross-sectional view of the patterning process of the passivation layer (mask: input/output pads; this process will be ignored if the wet-etching post-process for removing sacrificial metal ME1~ME6 layers is needed). The process flow of TSRI CMOS MEMS foundry [4]: (**d**) the cross-sectional view of coating the thick photoresist layer. (**e**) The cross-sectional view of the patterning process of the thick photoresist layer (mask: MEMS_OPEN). (**f**) The cross-sectional view of the RIE/anisotropic etching process on passivation and oxide after removal of the ME7 layer while the α-Si layer remains. The process flow of the TSRI CMOS MEMS foundry [4]: (**g**) the cross-sectional view after stripping the thick photoresist layer while the α-Si layer remains. (**h**) The cross-sectional view of starting the XeF_2_ isotropic silicon etching while the α-Si layer remains. (**i**) The cross-sectional view of the final released CMOS MEMS structure after removal of the α-Si layer with a 45 μm-deep cavity underneath the MEMS_OPEN area.

Since 2019, CIC and Nano Device Lab—NDL have officially merged into TSRI to accelerate the CMOS MEMS platform maturation. UMC provided a 0.18 μm CMOS MEMS process, combining the UMC 0.18 μm 1P6M mixed-signal/RF CMOS process with the MEMS post-process of trench RIE and XeF_2_ etching shown in the process flow of Figure 2. This UMC CMOS MEMS process is now applicable to the fabrication of MEMS devices such as suspension beams, perforated membranes [6,7], accelerometers, gyroscopes, and so on. It also aligns with the final dream that CMOS MEMS designers in the future can play a similar role as the fabless IC designers nowadays. In other words, the MEMS designers need not have their own post-processing facilities but will receive packaged MEMS chips after their tape-out by the CMOS MEMS foundry service provided by TSRI.

By the aforementioned CMOS-MEMS foundry service in Taiwan, some capacitive sensing, environmental sensing, and system-level integration applications were made. Hsieh et al. developed CMOS-MEMS capacitive tactile sensors with vertically integrated electrode structures, achieving enhanced sensitivity through stacked CMOS metal layers [23]. Lee et al. further demonstrated CMOS-MEMS technologies for environmental sensing applications, integrating multiple sensing functions such as pressure, infrared, and gas sensing within a single CMOS-compatible platform [24]. The CMOS-MEMS-based sensor integration for multi-parameter sensing and compact system design, highlighting the capability of CMOS platforms to support highly integrated sensing hubs with low power consumption and high reliability, were ever done [25]. These studies collectively indicate that CMOS-MEMS technology in Taiwan has progressed toward integrated and scalable sensing platforms, supported by standardized post-processing techniques consistent with the TSRI CMOS-MEMS approach.

In addition to CMOS MEMS development in Taiwan, the accelerometers obtained by isotropic oxide etching of the CMOS back-end of line (BEOL) stack in IHP SG25 technology were taped out and demonstrated improvement [26]. Narducci et al. demonstrated CMOS-MEMS capacitive pressure sensors fabricated using standard CMOS processes, confirming the feasibility of monolithic integration between mechanical sensing structures and on-chip readout circuits [27]. Furthermore, recent developments in CMOS-MEMS resonators and integrated systems have shown that multilayer CMOS structures can be effectively utilized to realize high-performance microstructures with strong electromechanical coupling [28]. More recent studies have also reported CMOS-MEMS devices with integrated front-end circuits, enabling improved signal processing and compact system-level design suitable for modern sensing applications [29].

These works collectively demonstrate the evolution of CMOS-MEMS technology from individual device fabrication toward highly integrated, multi-functional sensing platforms, which align closely with the TSRI CMOS-MEMS process and its application to capacitive sensors and actuators. In view of the two technical issues with the previous CMOS MEMS processing platforms: (1) they often only produce specific types of MEMS devices and cannot accommodate both designs of sensors and actuators on the same CMOS chip; (2) designers still need to do a lot of post-processing work on their own using expensive clean rooms and processing facilities. Therefore, this paper specifically addresses the needs of capacitive sensors and electrostatic actuators, based on TSRI CMOS MEMS foundry services. Under the premise that MEMS designers are equipped with a very simple wet etching technique, it proposes adding minimal post-processing and chip packaging treatments to compensate for the incompleteness of the current TSRI CMOS MEMS foundry. It is hoped that in the future, TSRI might adopt the additional post-processing proposed in this paper, allowing MEMS designers to truly enjoy the services of a fabless MEMS foundry.

As for the reason this article limits itself to capacitive sensing and electrostatic actuation technologies, besides better compatibility with the CMOS process, it is also because of the potential future applications or extensions of CMOS MEMS in the space field [30]. The space field involves strict thermal insulation and thermal management, so the power consumption and heat generation of MEMS components must be minimized. Capacitive devices with less temperature drift effect perform better than other piezoresistive or piezoelectric transducers in this area, apparently.

Although TSRI CMOS-MEMS foundry services provide suspended microstructures and standard post-processing support, the current platform still has limitations for realizing more advanced capacitive sensors and electrostatic actuators requiring embedded capacitive gaps, sacrificial metal release, and localized post-processing. Therefore, a practical extension strategy compatible with simple laboratory facilities is still needed.

## 2. CMOS MEMS Process by TSRI

The following is the TSRI’s CMOS MEMS post-process description: The UMC 0.18 μm CMOS process is composed of one polysilicon (Poly) layer and six metal layers which can be used to compose the stacked microstructures as shown in Figure 2a. Following the CMOS process, an additional amorphous silicon (α-Si), metal 7 (ME7) layer and passivation layer are deposited and patterned as shown in Figure 2b. It should be noted that the ME7 mask represents the definition of both the thin films of α-Si and the ME7 layer. During the subsequent RIE and XeF_2_ post-processing steps, the ME7 layer is removed earlier, while the α-Si layer temporarily remains and is removed only in the final release stage. The ME7 mask is important to define the configuration of the free-standing MEMS structure geometry within the MEMS_OPEN region.

Regarding the etch-hole patterns designed for ME7, there are two types. The first type is square openings, with the opening size ranging from 8 µm to 12 µm and the spacing between openings ranging from 8 µm to 17 µm. The recommended combination of opening size and spacing is shown in Table 1. Areas densely filled with etching openings will eventually be processed into perforated suspended thin films.

The second type of ME7 etched mask pattern mainly supports the fabrication of fork-shaped comb microstructures with elongated rectangular grooves. The groove width varies from 1.5 µm to 5 µm, and the width of the fork bars varies from 3 µm to 7 µm. It is recommended that the width of the fork bars match the groove width, as shown in Table 2. For smaller pattern sizes, the final material of the fork bar structures is roughly the only metal remaining; for larger (>7 µm) non-fork bar structures using elongated rectangular grooves, suspended structures such as cantilever bridges/beams and blocks can be defined. (Please refer to the explanation in the section of comb-shaped resonators).

Figure 2c shows the full CMOS process with an etch-resistant passivation layer. The input/output pads were opened in this step if no more wet etching post-process other than the UMC MEMS post-process herein.

Subsequently, a thick photoresist layer, as depicted in Figure 2d, is deposited and defined over the circuit and other areas, except the MEMS region, for etch protection using the MEMS_OPEN mask, as shown in Figure 2e. The entire post-CMOS fabrication is performed using dry etching processes. Figure 2f illustrates the reactive ion etching (RIE) process on the silicon dioxide layers.

Please note that at this point, the passivation layer as well as the ME7 layer have already been removed during the RIE process, while the α-Si layer still remains. Because the suspended microstructure needs to remain flat after release without warping, the topmost layer of the CMOS stack should not include passivation silicon nitride materials with stress [12]. Additionally, to reiterate, this MEMS_OPEN step uses the previously patterned ME7 layer shown in Figure 2b as the masking structure during oxide etching to pattern the vertical etch holes and trenches within the MEMS_OPEN area; areas outside MEMS_OPEN, including IC regions and previously opened pads, are protected by the thick resist in this step and are not affected.

Figure 2g shows the structure after stripping the thick photoresist layer while the α–Si layer still remains on the top surface after the post-CMOS micromachining process and silicon dioxide etching.

Figure 2h shows the XeF_2_ isotropic silicon etching process while the α-Si layer still remains on the top surface of the structure, where the silicon substrate is isotropically etched by XeF_2_ without photoresist protection, while the IMD6 oxide layer acts as the etching stop layer, resulting in partial thinning of the passivation layer. (Note: “inter metal dielectrics—IMD” and “inter layer dielectrics—ILD” oxides and ME1~ME6 metal layers are not attacked by XeF_2_ gas-phase etchant.)

Finally, Figure 2i shows the fully released CMOS MEMS structure after removal of the α-Si layer. It is noteworthy that IMD6 persists on the ME6 layer and there is a 45 μm-deep cavity underneath the MEMS region or the MEMS_OPEN area.

In addition, residual stress effects were evaluated qualitatively based on the released CMOS-MEMS structures fabricated using the proposed process. No significant bending, curling, or warpage was observed in the released cantilevers and suspended structures. This behavior is mainly attributed to the removal of the passivation silicon nitride layer during the post-processing sequence, thereby reducing the influence of tensile residual stress on the final free-standing structures. Furthermore, the achievable aspect ratio of suspended structures is constrained by the CMOS layer thickness, release geometry, and etching conditions. Structures with excessively large length-to-thickness ratios may become more susceptible to deformation, stiction, and mechanical fragility during release; therefore, practical design guidelines should be considered when defining suspended MEMS structures.

In summary, the TSRI CMOS-MEMS process first defines the suspended CMOS geometry using the ME7 mask and MEMS_OPEN region. Subsequently, RIE removes the oxide layers and exposes the silicon substrate, followed by XeF_2_ isotropic silicon etching to release the suspended structures. Finally, optional wet etching of sacrificial metal layers enables the formation of embedded capacitive air gaps for capacitive sensing and electrostatic actuation.

The vertical etching hole sizes and spacings do not meet the recommendations of Table 1 and Table 2, so it cannot be guaranteed that the 45 µm-deep cavity under the CMOS stack will be continuously penetrated, resulting in local pillars that prevent the MEMS_OPEN area from being suspended. The remaining pillar structures that are not suspended are not useless; they can serve as the lower electrode external connection port of a capacitive sensor or an electrostatic actuator, known as an anchor (to be explained in the sections on accelerometer and torsional mirror, etc.) [31].

All of the above CMOS layer layouts were designed using Cadence IC 6.1.8 software licensed by TSRI and must pass the design rule check (DRC) before being sent to TSRI for fabrication.

## 3. MEMS Devices by TSRI’s CMOS MEMS Foundry

### 3.1. Perforated Membrane for Flow Sensors

**Figure 3:**
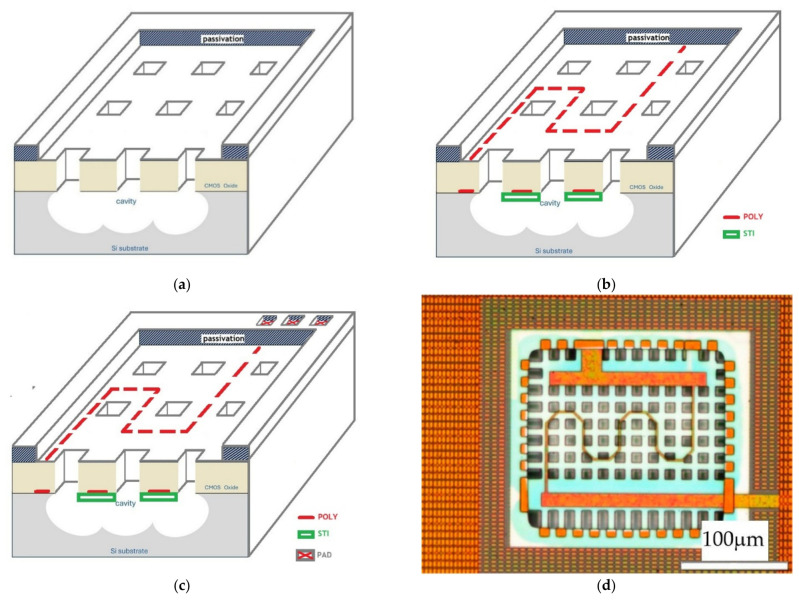
A uniform perforated membrane for a CMOS flow sensor with POLY RTDs: (**a**) 3D configuration for the membrane; (**b**) the membrane with STI to protect the POLY RTDs; (**c**) the open pad consideration; (**d**) the fabricated flow sensor [6,33].

Figure 3a is a uniform perforated membrane for a CMOS flow sensor with polysilicon resistive thermal detectors (RTDs) [6]. This is the first CMOS MEMS component configuration promoted by TSRI. As long as it meets the whole specification in Table 1, no additional post-processing etching is required, and the chip can be returned for testing directly.

The red dashed wire shown in Figure 3b represents POLY wiring or metal wiring, which bypasses the location of the vertical etched holes for use as the sensing site and its electrical connection. However, when using POLY wiring, because its original function is the transistor gate and it is located at the bottom layer of CMOS, extremely close to the silicon substrate, POLY can easily be removed by XeF_2_ during isotropic silicon etching, causing failure. Therefore, it is necessary to specify an additional shallow trench isolation (STI) oxide layer, placed “below” the POLY to provide protection during XeF_2_ etching. However, the positioning of this STI actually violates transistor design rules—the STI is usually placed ‘between’ transistors (i.e., at the POLY locations) to act as signal isolation, rather than ‘under’ the POLY. Therefore, the above approach requires approval from TSRI in order to be handled flexibly [32]. Furthermore, regarding the passivation layer photomask described in the aforementioned Figure 2c process, the openings for the output/input pads must be designed in advance; otherwise, after the CMOS MEMS chip fabrication is completed, wire bonding/packaging cannot proceed. The schematic diagram of the flow sensor example is shown in Figure 3c, and the photo of the completed flow sensor is shown in Figure 3d [33].

### 3.2. Capacitive Force/Pressure Sensors

**Figure 4:**
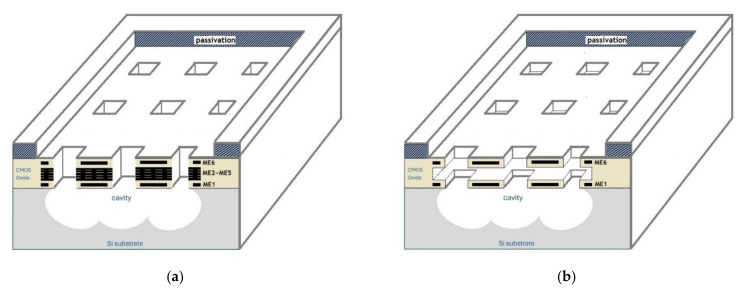

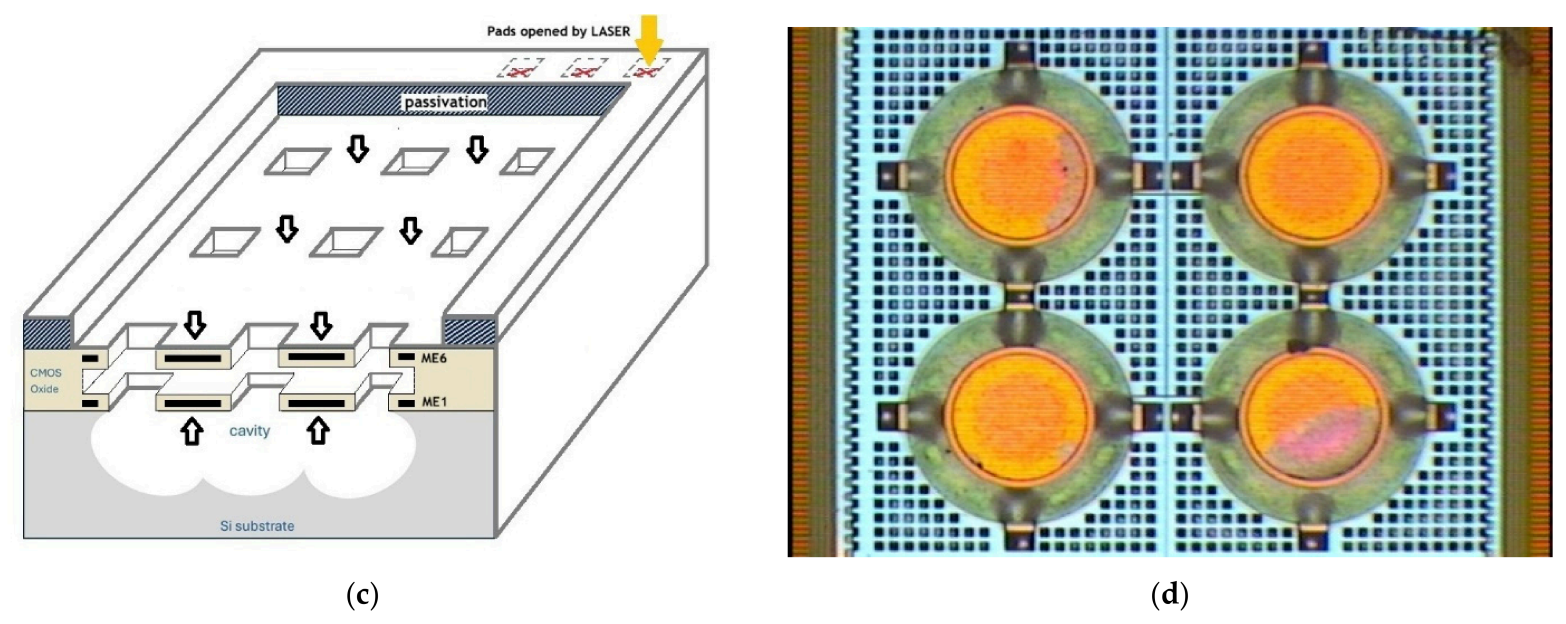
A non-uniform-thickness membrane for a CMOS capacitive chamber-type pressure sensor: (**a**) 3D configuration; (**b**) removing the metal sacrificial layers ME2~ME5; (**c**) subject to pressure and the shielded pad consideration; (**d**) the fabricated pressure sensor [7].

Figure 4a is a non-uniform-thickness membrane for a CMOS capacitive pressure sensor. At first glance, Figure 4a still maintains a uniform perforated thin-film structure similar to Figure 3a, but the photomask patterns of the ME2~ME5 metal layers, along with the related vias, are almost identical to the previous ME7 photomask pattern (with even some edges protruding slightly). The design of the ME2~ME5 metal layers does not particularly aid in the processing of vertically etched holes, but it is very important for the actual formation of capacitive sensors or electrostatic actuators.

Figure 4a differs from Figure 3a in the design of the CMOS metal layers because after completing the CMOS manufacturing process in Figure 4a, the designer can further perform wet etching on the metal sacrificial layers ME2~ME5 (simply by using a beaker with acid for etching, rinsing, and drying; a cleanroom is not even necessary). Since the patterns of the metal sacrificial layers ME2~ME5 slightly extend beyond the ME7 photomask patterns, the edges of ME2~ME5 can actually be seen exposed on the inner walls of the vertical etch holes. Thus, when the metal etchant is introduced, many of the interlayer metal sacrificial layers ME2~ME5 are eventually removed, leaving the lower electrode ME1 and upper electrode ME6, which were originally encapsulated by the interlayer dielectric (ILD), forming a capacitor pair. The spaces left after the exposed ME2~ME5 are etched away, becoming the air gaps of the capacitor (4.72 μm according to the thickness information of all CMOS layers provided by TSRI), as shown in Figure 4b. If a surface traction force is applied, compressing the capacitive air gap changes the capacitance value, which constitutes a capacitive force sensor; or after parylene seals and the input/output pads opening, it functions as a capacitive pressure sensor [31], where applied pressure changes the air gap and capacitance value. Conversely, if a voltage difference is applied between ME1 and ME6, generating electrostatic attraction between the upper and lower electrodes, reducing the air gap distance, and even causing a pull-in phenomenon [34], the hollow thin film can achieve vertical “squeeze (pressure) recovery” braking, which is regarded as a type of electrostatic actuator.

Figure 4b shows that the capacitive gap is extremely small, only a few micrometers. During the process of removing the metal sacrificial layer with acid and during cleaning and drying, capillary adhesion can easily occur [34]. Supercritical CO_2_ drying (Tousimis, SAMDRI-795, Tousimis Research Corporation, Rockville, MD, USA) must be applied to prevent stiction failure [31].

In addition, in response to large-area MEMS suspended structures, which may break or be damaged due to liquid impact during the wet etching process, it is possible to additionally consider adding reinforced tethered beams. This part will be explained in the text of Section 3.7.

Because the post-process involving acid corrosion will also attack the exposed electrodes of the output/input pads, the passivation openings at the pad locations in Figure 2c must also be temporarily skipped (do not draw pad opening patterns on the passivation mask) so that the passivation continues to protect the pad metal from corrosion. After all processes are completed and before wire bonding and packaging, the passivation above the pad is locally ablated using a laser (such as the machine provided for external service by TSRI, Azoom2 (Polytec GmbH, Waldbronn, Germany), New Wave Research, burst-mode laser wavelength 532 nm) to complete the pad opening process, as shown in Figure 4c. This step is also a supplementary measure corresponding to the post-process wet etching of the added metal sacrificial layer in this subsection and was first proposed by the author’s team for the development of CMOS MEMS devices. This laser-assisted pad-opening approach provides a practical solution for protecting aluminum pads during sacrificial wet etching and is especially useful for CMOS-MEMS devices requiring additional laboratory-level post-processing. Figure 4d is a physical photo of the completed CMOS capacitive pressure sensor [7].

### 3.3. Capacitive Cantilevers and Accelerometers

**Figure 5:**
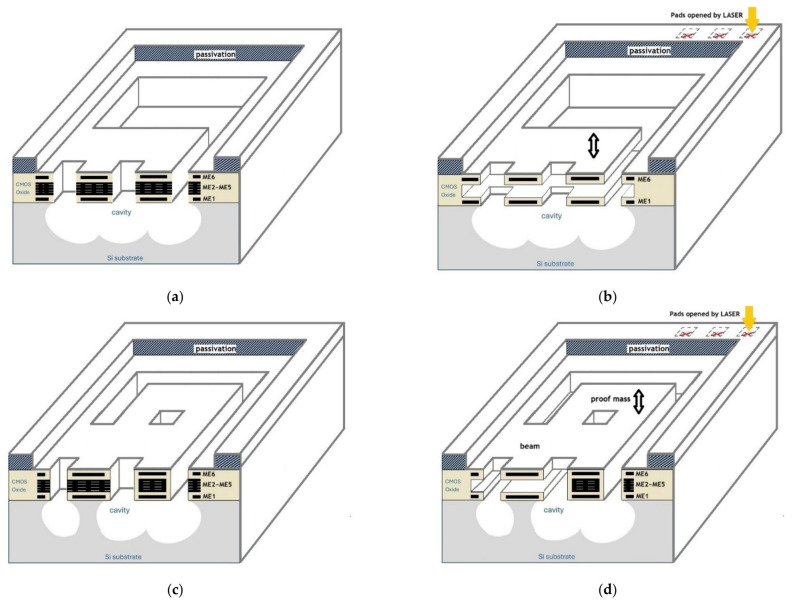
Capacitive cantilevers and accelerometers: (**a**) 3D configuration of a cantilever; (**b**) removing the metal sacrificial layers ME2~ME5 for the cantilever; (**c**) 3D configuration of a beam with a proof mass for a CMOS capacitive accelerometer; (**d**) removing the metal sacrificial layers ME2~ME5 for the accelerometer subject to vertical acceleration.

Following the CMOS capacitive pressure sensor in Figure 4, by modifying the ME7 mask and designing the corresponding metal sacrificial layer, and then using the same CMOS MEMS post-processing along with designer-performed wet metal etching, one can fabricate the CMOS capacitive cantilever or capacitive accelerometer shown in Figure 5 for single-axis inertial force measurement along the chip plane’s normal direction.

Figure 5a shows a simple cantilever-shaped capacitance electrode pair. The designer further performs acid corrosion on the ME2~ME5 metal sacrificial layers, which can reveal the capacitance electrode air gap, as shown in Figure 5b.

In order to increase the measurement sensitivity of the inertial force, the shapes of the “proof mass” and the “beam” must be clearly defined. Therefore, the cross-section of the capacitive accelerometer in Figure 5c shows that the right end retains the CMOS metal layers and is wrapped within the ILD oxide layer, enlarging and adding weight to the “proof mass.” On the left side, the metal layers ME2~ME5 are removed, leaving the ILD oxide layer surrounding ME6 as the “beam,” which serves as the upper capacitor electrode ME6, in order to reduce the beam stiffness. At the same time, increasing the mass and reducing the beam stiffness will effectively lower the natural frequency of the overall “mass-beam” structure, which is beneficial for capturing acceleration signals that generally fall within the low-frequency range.

The displacement of the mass block vibration is represented by the ‘thin beam,’ which means the upper electrode ME6 of the capacitor acts as the vibrator; the lower electrode ME1 of the capacitor remains connected to the silicon substrate at the ‘anchor’ and acts as the stator. The formation of this anchor relies on reducing the size of the vertical etching holes and increasing the spacing between the holes (refer to Table 1) so that the 45 μm deep cavity cannot laterally connect to a neighboring etched hole cavity; thus, the lower electrode ME1 of the capacitor remains fixed to the silicon substrate, similar to the posture of a mushroom. However, after the wet acid etching removes ME2~ME5 to form the capacitor gap, the upper and lower electrodes of the capacitor separate, and the lower stator does not interfere with the suspension and vertical axis vibration of the upper mass block and thin beam. The CMOS accelerometer designed in accordance with the concept described in this paragraph would have the 3D geometric structure as shown in Figure 5d upon completion.

### 3.4. Capacitive Comb-Shaped Resonators or Comb Drives

**Figure 6:**
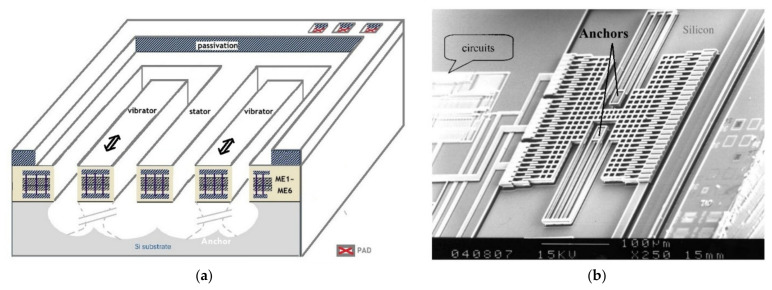
A CMOS interdigital comb structure with vibrators moving in-plane subject to horizontal acceleration or electrostatic driving: (**a**) 3D configuration; (**b**) a CMOS prior art [12].

The capacitive sensors in Figure 4 and Figure 5 sometimes incorporate electrostatic force feedback to maintain the capacitor electrodes near the central balanced (zero displacement) position in order to avoid nonlinear output caused by excessive changes in the capacitor air gap, thereby improving output linearity. However, capacitive sensors do not necessarily have to rely on the capacitance value changing inversely with the air gap; they can also change in proportion to the capacitor area. For example, the comb drive for horizontal vibration in Figure 6 (direction of back-and-forth vibration, see the double-arrow symbol in Figure 6) changes capacitance not because of inverse variation in the air gap distance, but due to proportional changes in the overlapping area of the stator and resonator, so the sensing is inherently less prone to nonlinearity issues.

This is also why the accelerometer design in the previous section for measuring the normal direction of the chip plane is not as common as the comb drive in Figure 6; therefore, this section introduces how to design a CMOS comb-shaped resonator or comb drive. This is also the second type of CMOS MEMS component configuration promoted by TSRI. As long as the design dimensions comply with the groove specifications in Table 2, the CMOS MEMS flow sensor in Figure 3 can be tested immediately after the chip is returned without any additional post-etch processing.

**Figure 7:**
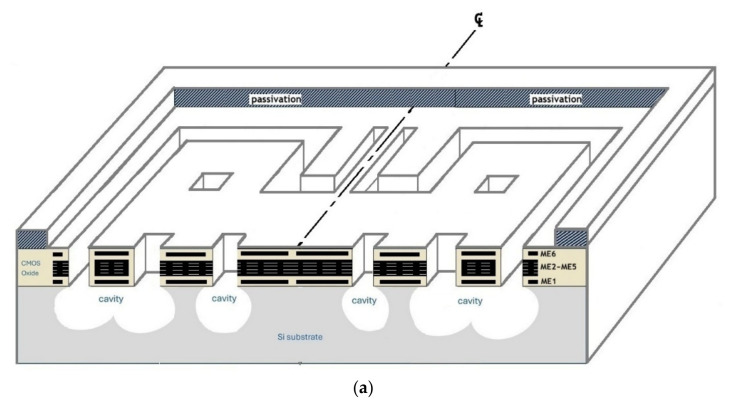

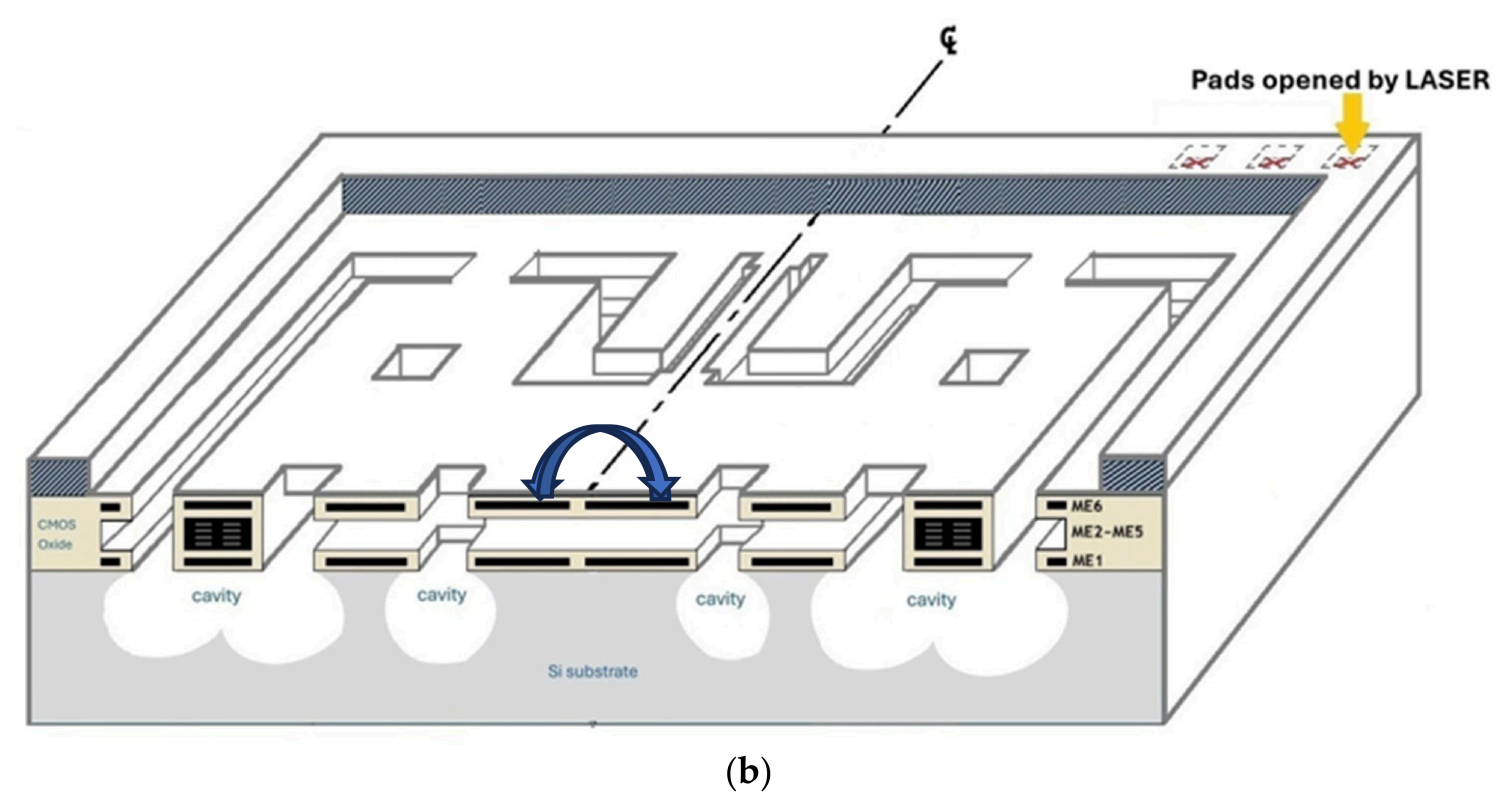
A DMD-like mirror pair with a torsional beam: (**a**) 3D configuration; (**b**) removing the metal sacrificial layers ME2~ME5.

Compared to the CMOS MEMS devices using hole-type suspended thin films as the substrate in Figure 3, Figure 4 and Figure 5, Figure 7a shows a 3D cross-sectional view of CMOS fork-shaped microstructures, indicating that during the mask design of ME7, long strip-shaped etching micro-grooves replaced the role of most micro-holes, especially in the areas where the stator and the vibrator are alternately interdigitated (sensing or driving parts) and the regions of slender spring deformation structures. To ensure a uniform horizontal electric field, the metal layers ME1~ME6 embedded between the ILD oxide layers need to fill as much as possible the vertical inner wall surfaces of the CMOS structural layers inside the micro-grooves, with the stator and vibrator positioned opposite each other in pairs.

The stator in Figure 6a has already been fixed and suspended in the frame, so there is no issue; however, after the suspended vibrator passes through the slender spring deformation structure with several bends, the question is where it is finally fixed. The answer is the ‘anchor.’ The layout and position of the anchor in the comb drive structure design can be referenced from the implementation example in Figure 6b [12].

With the experience of designing and fabricating the CMOS MEMS fork-shaped capacitive accelerometer at this location, the design can be extended to the component design of a fork-shaped capacitive gyroscope (gyro) [35]. Its CMOS MEMS post-processing is similar to that of the accelerometer shown in Figure 6 and will not be described here.

As mentioned in the case of the Figure 3c flow sensor, the designer must ultimately pay attention to the fact that the photomask for the passivation protective layer described in the Figure 2c process instructions must be designed in advance with openings for the input/output pads; otherwise, wire bonding and packaging cannot be directly performed after the CMOS MEMS chip processing is completed.

### 3.5. Capacitive Torsional Mirrors

One of the fundamental difficulties in designing electrostatic actuators is the limited actuation stroke [34]. Therefore, to increase the actuation stroke, it is necessary to make good use of instabilities such as electrostatic pull-in and continuously increasing deformation. The famous Digital Light Processing (DLP) digital mirror display (DMD) by Texas Instruments [36] is a successful example. Here, an attempt is made to use TSRI’s CMOS MEMS foundry process to replicate a DMD unit as shown in Figure 7.

Figure 7 shows the mirror pair structure, supported only by a torsional beam. Because there is no bearing design, this torsional beam supports the entire mass of the suspended mirror pair and also allows the mirrors to twist; meanwhile, the wires connecting to the upper electrodes of the external capacitors of the suspended mirror pair also pass through and are laid along this slender torsional beam. As for the lower electrodes of the capacitors and their metal wiring, they are fixed to anchor points on the silicon substrate and do not twist with the mirror structure.

Figure 7a depicts the 3D configuration of the DMD-like mirror pair with a torsional beam. Figure 7b shows the free-standing mirror pair after removing the exposed ME2~ME5 sacrificial metal layers. The hole spacing of the free-standing structure and the anchor region is different. According to Table 1, if the etch-hole size is selected as 8, 9, 10, 11, or 12 μm, the hole spacing of the free-standing structure should be within 10, 11, 13, 14, or 15 μm, respectively. Meanwhile, the hole spacing of the anchor region should be larger than 12, 13, 15, 16, or 17 μm, respectively.

The cross-section of the torsional beam in Figure 7 should be a square so as to be close to a circular bar to achieve more stable torsional motion. In other words, the width of the torsional beam is supposed to be equal to the thickness of the ME6 metal and the laminated IMDs. Additional geometric reinforcement near the torsional beam anchor regions may also be required to reduce stress concentration and improve structural reliability during repeated torsional operation. For optimizing the device dimensions, the static stress/deformation finite-element-analysis (FEA), the modal FEA of the device’s dynamic behavior, the electrostatic field/force FEA, and the critical wetting area evaluation at the capacitive gaps due to the surface tension during the wafer drying process [37], etc., are all necessary in the device design stage.

### 3.6. Scratch Drive Actuators (SDAs) with Reset Function and Adding Dimples Against Surface Stiction

**Figure 8:**
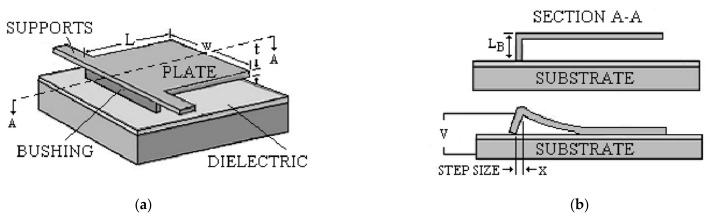

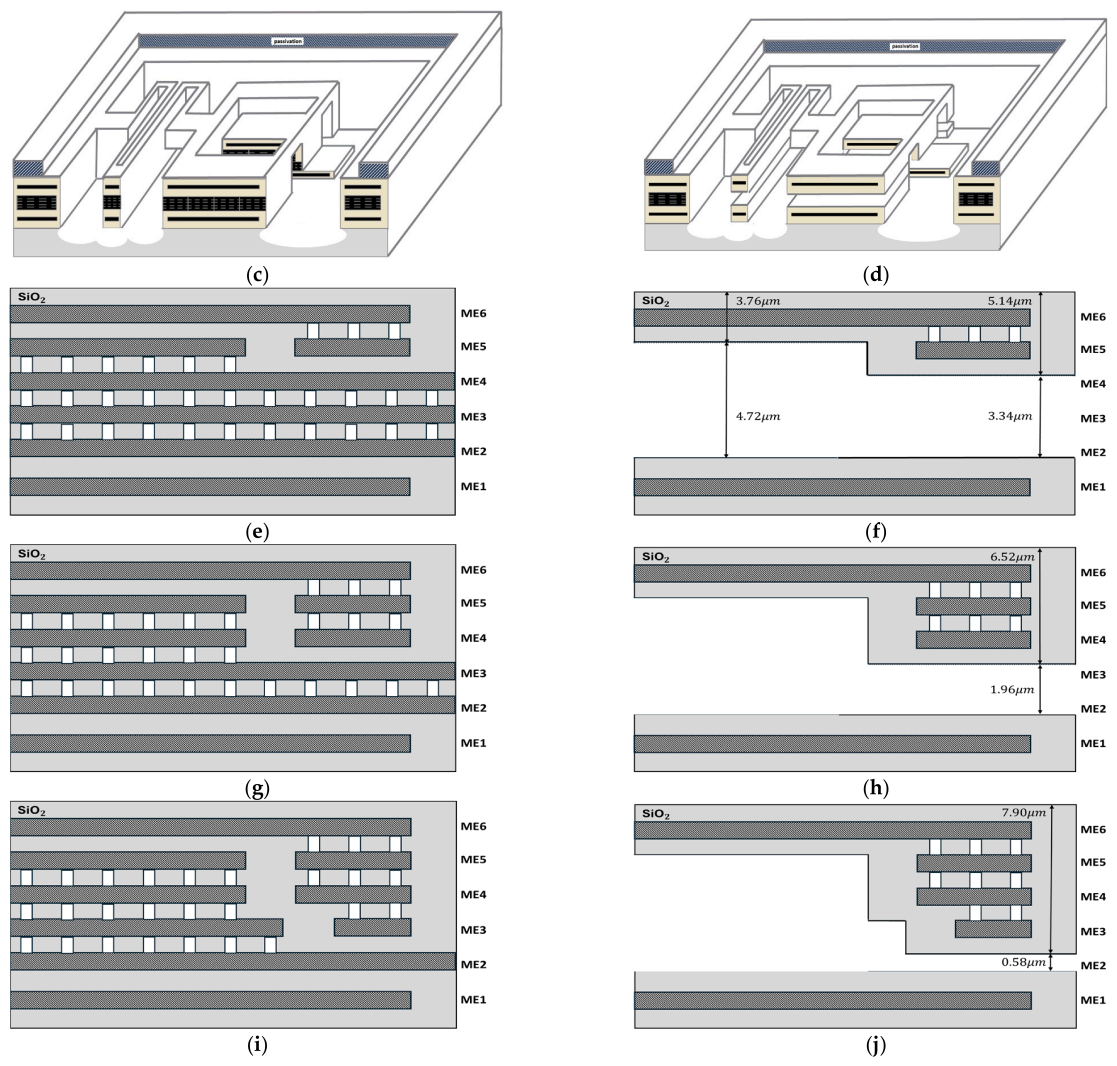
A SDA: (**a**,**b**) schematic and actuation principle of SDA [39]; (**c**) 3D configuration; (**d**) removing the metal sacrificial layers ME2~ME5; (**e**,**g**,**i**) different layer designs of the bushing structures; (**f**,**h**,**j**) the different bushings after releasing.

Continuing the spirit of the torsional beam, allowing electrostatic micro actuators to generate larger travel strokes, another electrostatic micro actuator that applies the torsional beam and has a simple structure is the scratch drive actuator (SDA) [38]. SDA, shown as Figure 8a,b mimics the movement of a caterpillar. After applying a cycle of attracting and releasing electrostatic force, the L-shaped bushing structure at the front end of the SDA bends downward and moves forward a short distance. When a square-wave-like driving voltage is applied and enough actuation cycles are accumulated, the SDA will also accumulate a noticeable forward distance [39].

SDA has been applied to the alignment and assembly of optical MEMS components [34]. In Figure 8c,d, the SDA actuation unit is fixed to the C-shaped frame with a torsional beam, and the frame is then fixed to the leftmost end of the chip component with a multi-bent flexible spring. The flexible spring is used firstly as an anchor for the SDA suspended structure and secondly to provide the SDA with a reset action after actuating. That is, when the zeroing drive voltage is applied, the spring can automatically pull the SDA back and restore it to its initial position.

The bushing structure at the front end of the SDA, taking the design in Figure 8c,d as an example, requires the SDA main plate and its bushing to be stacked to achieve different thicknesses. Assuming the fixed thickness of the SDA main plate as the ME6 layer along with the IMD oxide layers above and below it, totaling 3.76 μm unchanged. After the lower ME2~ME5 metal sacrificial layers together with the tungsten via are removed, resulting in a 4.72 μm capacitive air gap that also remains unchanged, too. However, for the bushing, one can choose the composite layer of ME5 and ME6 as in Figure 8e, the composite layer of ME4~ME6 as in Figure 8g, or the composite layer of ME3~ME6 as in Figure 8i, corresponding to three thickness combinations. The greater the thickness difference between the plate and the bushing, the better the braking effect of the SDA moving forward.

Of course, if the bushing thickness increases from 5.14 μm in Figure 8e up to 7.90 μm in Figure 8i, the underlying capacitor air gap must also decrease from 3.34 μm down to 0.58 μm. The 0.58 μm air gap is very likely for the upper and lower electrode surfaces to be pulled into contact by liquid capillary forces during the drying process after wet etching. However, if the actual projected area of the bushing is much smaller than the projected area of the SDA main plate, differing by one or two orders of magnitude, the bushing contact area will conversely act like a dimple or post structure that prevents adhesion from capillary forces [40].

### 3.7. Adding Tethered Beams Against Surface Stiction

The MEMS post-processes provided by TSRI all use dry etching (including RIE and XeF2 gas etching), which naturally avoids the issue of capillary liquid adhesion. However, the acid-etching metal sacrificial layer post-processes specifically added for capacitive sensors or electrostatic actuators, as suggested in this article, do have the issue of capillary liquid adhesion. To prevent MEMS suspended structures from sticking to the substrate or even being prematurely pulled apart by capillary forces/surface tension of liquids, Section 4 of this article recommends using supercritical carbon dioxide drying as a preventive measure. Section 3.5 also mentions that during device size optimization, it is suggested to pre-evaluate the critical wetting area as a second line of defense.

**Figure 9:**
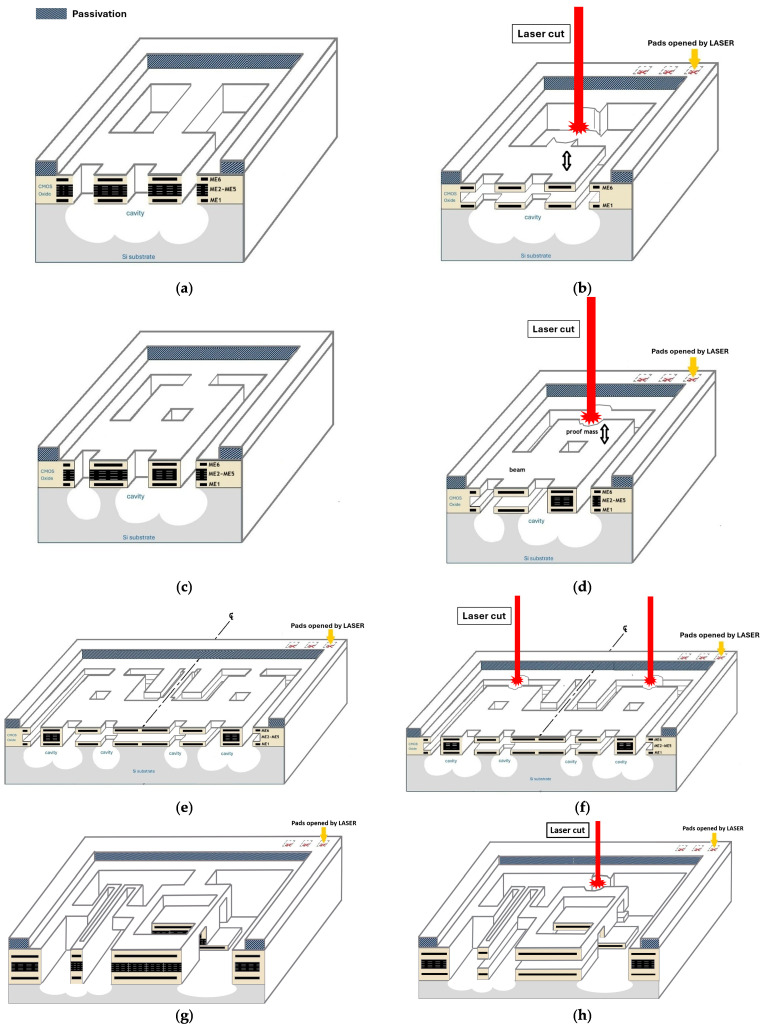
Addition of tethered beams against surface stiction: (**a**) strengthen the original Figure 5a design; (**b**) after removing the metal sacrificial layer from (**a**) to create suspension, use a laser to remove the tethered beams; (**c**) strengthen the original Figure 5c design; (**d**) after removing the metal sacrificial layer from (**c**) to create suspension, use a laser to remove the tethered beams; (**e**) strengthen the original Figure 7a design; (**f**) after removing the metal sacrificial layer from Figure 7b to create suspension, use a laser to remove the tethered beams; (**g**) strengthen the original Figure 8a design; (**h**) after removing the metal sacrificial layer from (**g**) to create suspension, use a laser to remove the tethered beams.

If the aforementioned two methods cannot ensure the successful suspension of the capacitive MEMS device, the final approach proposes adding a tether reinforcement structure, that is, first ‘binding tightly’ the suspended parts of the MEMS to minimize deformation; after all wet corrosion post-processes are completed and the device is dried gently, continue with pad opening, MEMS chip wire bonding and packaging, and finally use a laser (Azoom2, New Wave Research, burst-mode laser wavelength 532 nm) to burn through the tether structure point by point, fully releasing the MEMS device, as shown in Figure 9.

## 4. Discussion

### 4.1. Acid Wet Etching

Table 3 lists the CMOS MEMS post-processing steps, with (B)~(K) suggested in this paper, added after TSRI’s dry etching post-processing.

The wet etching procedures in steps (D) and (E) only require preparing small beakers and wet etching solution and can be carried out in a generally air-conditioned laboratory; because the CMOS MEMS chip sizes are tiny, a large amount of etching solution is not needed. Therefore, there is little problem with waste liquid recovery.

The main function of the hydrochloric acid in step (D) is to remove the exposed aluminum sacrificial layer. During the chemical reaction, violent hydrogen gas bubbles are generated. The escape of these bubbles also facilitates the physical exchange between the wet etching reactants and products, so the reaction only takes a few minutes and does not require heating and agitation. As for the piranha solution in step (E), its main function is to remove the tungsten metal layer, organic matter, and residual metal debris. The piranha solution automatically heats above 100 °C during preparation, so no additional heating is required, either. The etching time depends on factors such as the aspect ratio of the capacitor air gap and the etching depth.

After completing the post-wet etching, the CMOS MEMS chips are still soaked in deionized (DI) water, then replaced with IPA organic solvent, and then sent to step (F) for supercritical carbon dioxide drying. Step (G) involves sending the CMOS MEMS chips to TSRI for die attachment, loading them into DIP packages, which facilitates handheld DIP package handling to continue the laser opening of input and output contact pads in step (G). Step (H) is the wire bonding. Step (I) involves outsourcing parylene coating. Step (J) returns to TSRI for laser cutting of tethered beams, and finally, after completing all post-processing, the CMOS MEMS chips enter the device electrical testing phase.

### 4.2. Laser Cutting

In this work, laser-based post-processing is considered an important supporting technique for CMOS-MEMS capacitive devices after wet sacrificial release. Similar to the laser ablation approach reported in [42], the laser system can be utilized for localized passivation opening, tether beam cutting, and selective structural modification without introducing excessive mechanical stress to suspended MEMS structures. Compared with conventional photolithography-based reopening processes, laser-assisted processing provides a simpler and more flexible method suitable for prototype-level CMOS-MEMS research and low-volume fabrication environments. This capability is particularly useful for capacitive pressure sensors, cantilever structures, and electrostatic actuators requiring additional post-fabrication structural release and electrical pad exposures.

**Figure 10:**
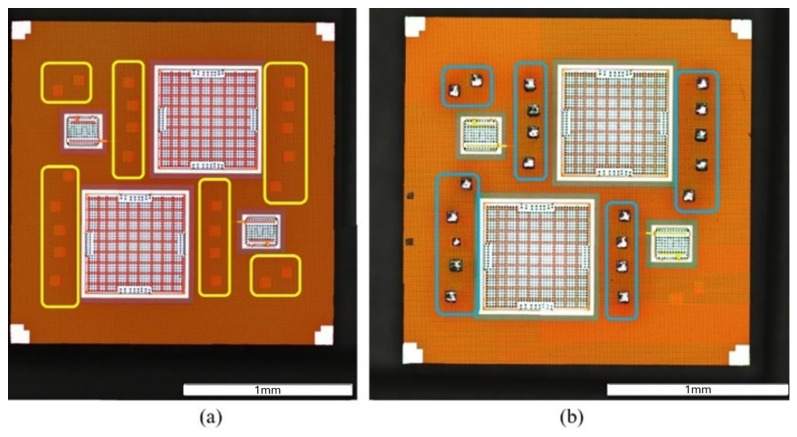
The pad opening done by the Azoom2 laser cutter: (**a**) before laser cutting; (**b**) after the laser cutting.

The suggested thickness of the tethered beams in Section 3.7 has two preliminary options: (1) ME6 with its surrounding IMD oxide layers, totaling approximately 3.76 μm; (2) ME5 + ME6 with their surrounding IMD oxide layers, totaling approximately 5.14 μm. For illustrations of these two thicknesses, see Figure 8e–j. The 3.76-μm thickness has lower stiffness, making it less resistant to downward deformation due to capillary forces. But it can be easily cut by point laser ablation using the Azoom2 laser provided by TSRI (New Wave Research, laser wavelength 532 nm). This laser cutting process is similar to the input/output pad opening in Figure 10, and it has been experimentally verified as feasible. As for the thicker 5.14-μm tethered beam, it has greater stiffness and better resistance to downward deformation due to capillary stiction, but the power of the Azoom2 laser needs to be appropriately increased.

### 4.3. Parylene Coating

**Figure 11:**
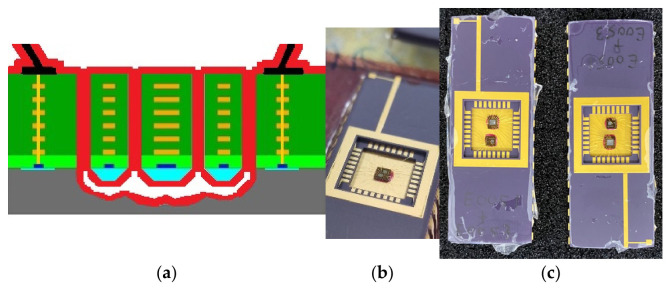
Parylene coating on the CMOS MEMS devices: (**a**) The cross section of MEMS chips conformally coated with parylene. 10 μm-thick parylene not only fills the 8 μm × 8 μm etch-holes and seals the pressure chamber, but also coats over the fine wires; (**b**) a DIP package with MEMS chips before parylene coating; (**c**) DIP packaged chips after parylene coating.

The room temperature (25 °C) vacuum coating process of parylene is illustrated in Figure 11a. This work is outsourced to Lachi Corp. [41]. The authors have coated parylene with thicknesses of 5 μm and 10 μm, respectively. It is found that 10 μm-thick parylene provides better sealing. It should be specially noted that the surface mobility of the parylene vacuum coating is excellent, allowing it to cover the chip surface, the outer regions of the aluminum bonding wires, and the vertical inner walls of the 8 μm × 8 μm holes. Parylene not only seals the pressure cavity but also effectively isolates the entire chip along with the bonding wires from the negative effects of external moisture and static electricity. The CMOS pressure sensor chip with a DIP package before and after the parylene coating is shown in Figure 11b,c.

**Figure 12:**
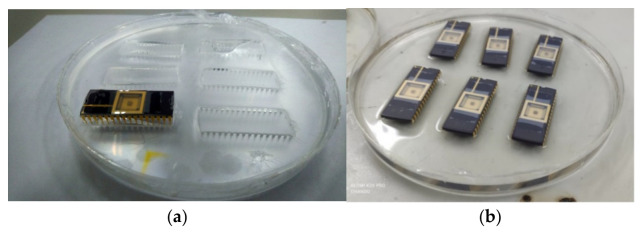
Pretreatment for DIP-packaged sockets MEMS chips before parylene coating: (**a**) a PDMS base molded by DIP package chips, waiting for installing DIP-packaged MEMS chips; (**b**) the PDMS base with MEMS chips ready for parylene coating.

Regarding the pretreatment before parylene coating in Figure 11, it is briefly described here. Because parylene coating is conformal and fills all gaps, the DIP-packaged socket pins after wire bonding will also be coated with parylene if not masked beforehand. That will result in unwanted circuit insulation. However, masking these pins must be nearly seamless (with voids smaller than 1 micron). Applying photoresist all over the pins is feasible for preventing parylene adhesion, but it requires removing parylene from the pins manually by scraping parylene with a blade. Therefore, PDMS, which is hyper-elastic and has a smooth surface, is good to be used as the molding substrate. Before the PDMS fully cures and dries, the DIP-packaged sockets are temporarily inserted into the PDMS surface. Once the PDMS substrate mold has completely cured, the DIP-packaged sockets are removed, leaving recessed holes for the DIP pins, as shown in Figure 12a. When parylene coating is needed, the pins of DIP-packaged sockets with MEMS chips can simply be inserted into the corresponding holes at the PDMS substrate mold, as in Figure 12b, allowing parylene coating to proceed. The final product has DIP-packaged socket metal pins without parylene, while all other areas are coated with parylene conformally.

### 4.4. Anti-Stiction and Self-Sealing

The adhesion problem only occurs in the post-wet etching process proposed in this article in order to remove the metal sacrificial layer and obtain the capacitor air gap. For examples of pressure sensors: in Figure 4, accelerometers in Figure 5, torsional mirrors in Figure 7, and SDAs in Figure 8. For devices with consistent capacitor air gaps, directly comparing the so-called critical wetting area $A_{Q}$ in Equation (1) is easy to evaluate the feasibility [37]. Meanwhile, if there exist two or more kinds of capacitor gaps shown in Figure 8f,h,j, the total surface tension *F* was composed of more than two capillary sources of $p_{i}A_{i}$ as well [31]. In Equation (2), it assigned *i* = 2.

(1) $$A_{Q}<\frac{k\cdot z_{0}^{2}}{8\gamma \cdot cos\theta }$$

(2) $$F=p_{1}A_{1}+p_{2}A_{2}=2\gamma \cdot cos\theta (\frac{A_{1}}{z_{1}}+\frac{A_{2}}{z_{2}})$$

where γ is the liquid surface tension; θ is the contact angle; $p_{i}$ is the Laplace pressure drop of the area *i*; $A_{i}$ is the wetting area *i* with respect to gap $z_{i}$; *k* is the structure stiffness.

However, the primary capillary force is still largely contributed by the smallest gap region, assuming that the area of each region *Ai* is roughly equal. To reiterate, if the area of the smallest gap is not large enough, such as the aforementioned bushing of SDAs and the dimple/post in Section 3.6, even if some local adhesion occurs, the overall capillary force may still be too weak to overcome structural force *kz* (*z* is the structural deformation). Therefore, ultimately the entire structure will not get stuck, achieving the overall effect of preventing surface-tension adhesion.

Conversely, if the area of the minimum air gap section is too large and violates Equation (1), the overall structure will inevitably get stuck in the region of the minimum air gap. For example, in references [7,43], although the adhesion area is designed at the outer edge of the pressure membrane and at the inlets/outlets of microchannels or chambers, the remaining areas with larger air gaps remain suspended. Therefore, the adhesion sealing there actually provides an additional self-sealing function. If this can be cleverly utilized, the parylene coating in Step I of Table 3 can be effectively omitted, thereby simplifying the post-processing of CMOS MEMS.

## 5. Conclusions

This article presents the 0.18-μm CMOS MEMS foundry service platform provided by the TSRI in detail. For MEMS designers with simple wet etching equipment, the development of capacitive sensors and electrostatic actuators with minimal post-processing and chip packaging steps was shown. They include CMOS MEMS devices such as pressure sensors, accelerometers, micro mirrors, and scratch drive actuators. It is hoped that in the future, TSRI will have the opportunity to adopt the additional post-processing and packaging procedures proposed herein, allowing MEMS designers to truly enjoy fabless foundry services for MEMS without requiring their own wafer fabrication facilities. Although the proposed post-processing strategy enables the realization of various capacitive sensors and electrostatic actuators using the TSRI CMOS-MEMS platform, several practical limitations remain, including wet-etch uniformity, stiction risks, packaging complexity, and dependence on additional laboratory-level post-processing steps.

## Figures and Tables

**Table 1:** Recommended etch-hole size (μm) to release the suspended microstructures vs. the hole spacing (μm) [4].

	Hole Spacing	8	9	10	11	12	13	14	15	16	17
Hole Size											
8		O	O	O	Δ	×	×	×	×	×	×
9		O	O	O	O	Δ	×	×	×	×	×
10		O	O	O	O	O	O	Δ	×	×	×
11		O	O	O	O	O	O	O	Δ	×	×
12		O	O	O	O	O	O	O	O	Δ	×

O: full release; Δ: critical release; ×: not sure.

**Table 2:** Recommended bar size (μm) to release the suspended comb structures vs. the bar spacing (μm) [4].

	Bar Spacing	5	4	3	2.5	2.3	2	1.5
Bar Size								
3		O	O	O	O	O	Δ	×
4		O	O	O	O	O	Δ	×
5		O	O	O	O	O	Δ	×
6		O	O	O	O	Δ	×	×
7		O	O	O	Δ	×	×	×

O: full release; Δ: critical release; ×: not sure.

**Table 3:** The article suggests adding CMOS MEMS post-processing steps after the TSRI dry etching post-process in this article.

No.	Post-Process Step	Venue
A	TSRI CMOS MEMS fabrication by dry etching	TSRI
B	Chip delivery	TSRI
C	Initial inspection by optical microscope	Designer
D	HCl post-wet etching	Designer
E	H_2_SO_4_ + H_2_O_2_ (piranha) post wet etching	Designer
F	Supercritical CO_2_ drying	Designer
G	Pad opening by A-Zoom2 laser system	TSRI
H	Packaging/wire bonding	TSRI
I	Parylene coating (option for pressure sensors)	Lachi Corp. [41]
J	Tethered beam cut by A-Zoom2 laser system	TSRI
K	Data acquisition and device characterization	Designer

## Data Availability

The original contributions presented in this study are included in the article. Further inquiries can be directed to the corresponding author.
